# Knowledge and attitude among the mothers of children in age group 1–5 years regarding childhood hearing loss

**DOI:** 10.1371/journal.pone.0357285

**Published:** 2026-09-18

**Authors:** Anushree Bhagavan, Adithya Sreedharan Sanitha, Dhanshree Rajesh Gunjawate, Rohit Ravi

**Affiliations:** Department of Audiology and Speech Language Pathology, Kasturba Medical College Mangalore, Manipal Academy of Higher Education, Manipal, India; Universiti Malaya Fakulti Perubatan: University of Malaya Faculty of Medicine, MALAYSIA

## Abstract

**Aims:**

The present study aims to explore the knowledge and attitudes among the mothers of children aged 1–5 years regarding childhood hearing loss, its risk factors, effects, and acceptance.

**Methods:**

A cross-sectional survey was conducted among mothers of children aged 1–5 years using convenience sampling. Data collection at a tertiary care hospital was conducted using a structured questionnaire that covered demographic details, knowledge of childhood hearing loss, attitudes towards hearing loss, intervention, and sources of information. Descriptive statistics were used to summarise continuous variables, while discrete variables were summarised using frequencies and percentages. Associations between education levels and knowledge and attitude items were examined using Fisher’s Exact test.

**Results:**

A total of 215 mothers participated in the study. The mean age of the mothers was 29.35 ± 4.85 years, and the mean age of the youngest child was 3.2 ± 1.2 years. Most mothers were aware that hearing loss could be congenital or late-onset. However, substantial gaps were noted in understanding of the causes of hearing loss and management options. Awareness of medical management options was higher than that of the use of rehabilitative services (42.3%). Awareness of middle ear infections among mothers was moderate, with more than 60% being aware of such infections and believing they can be prevented. Acceptance towards hearing screening was high (86.5%); however, acceptance of amplification devices was low (27.4%). Although the attitudes towards childhood hearing loss were largely positive, the attitudes towards rehabilitation procedures and inclusivity were negative. Maternal educational level was significantly associated with attitudes toward hearing rehabilitation and educational inclusion (p < 0.05) but not with knowledge-related items. More than 80% of mothers expressed an interest in learning about the causes of hearing loss, treatment options, and where to seek help. Clinics or health workers were identified as the most preferred sources of information.

**Conclusion:**

Mothers demonstrated a moderate awareness of childhood hearing loss, with substantial gaps in causes and management options, and negative attitudes towards rehabilitation procedures and inclusivity. Focused parental education programs and community-based awareness initiatives would help reduce stigma and overcome misconceptions.

## Introduction

Hearing plays a crucial role in early language acquisition and development, as well as in communication, cognitive development, and socio-emotional growth. The impact of hearing loss, especially in infants and young children, can lead to delays and deficits across these domains [1–3]. The critical period between 1 and 5 years of age is crucial, as neural plasticity, along with appropriate sensory experiences, enables rapid skill acquisition and learning. Neural plasticity enables auditory experiences to shape and enhance the pathways of speech and language development [4,5]. Even mild hearing loss can lead to delays in the development of speech and language skills, academic challenges, and a lack of vocational and employment opportunities later in life. This can further impact interpersonal relationships, overall well-being, quality of life, and one’s role in society at large.

It has been estimated that overall, 5% of the world’s population (430 million) suffers from disabling hearing loss and needs rehabilitation services, which also includes 34 million children. This burden is projected to increase, with one in every ten individuals expected to be affected by 2050 [6]. Early hearing detection and intervention programs have accelerated the screening, diagnosis, and management of children diagnosed with hearing loss [7]. Nevertheless, despite such global efforts, delays in identification and intervention continue to occur in several regions due to inadequate follow-up [8,9], late-onset or progressive hearing loss [10,11], and a lack of awareness among healthcare professionals [12–14] and among parents of young children [15–17].

Mothers are frequently the primary caregivers for young children and play a pivotal role in observing and recognizing early signs and concerns, seeking medical services, and making decisions about treatment options. Consequently, their knowledge and attitude towards childhood hearing loss can have a substantial influence on the timely identification, diagnosis, and intervention for hearing loss. Considerable attention has been directed towards parental awareness in the neonatal period and newborn hearing screening. Limited knowledge and awareness, negative attitudes, misconceptions, and traditional and cultural belief systems have been identified as some of the reasons for delays and shortcomings in the success of such programs [16,18]. Previous studies from diverse settings have indicated that community education and caregiver-focused training can improve recognition of late-onset and acquired hearing loss, and facilitate identification of hearing loss in children who were not screened at birth [19].

The age range from 1 to 5 years is a critical period for auditory, speech, language, cognitive, and social development. During this period, children are at risk of late-onset hearing loss, progressive hearing loss, delays in speech and language development, and conductive pathologies especially involving the middle ear. If left unidentified and untreated, these can have long-term consequences for developmental, educational, language, and psychosocial outcomes. Children may develop late-onset, progressive, or acquired hearing loss after passing the newborn hearing screening, thereby highlighting the importance of continued hearing testing in children [20].

Previous studies investigating parental knowledge and attitudes have primarily focused on newborns and infants [15–17]. The concerns and challenges encountered by mothers of children aged 1–5 years differ considerably from those of mothers of newborns, particularly regarding diagnosis, rehabilitation, educational planning, and long-term developmental outcomes. This represents an important gap in the literature, as little is known about the mothers’ knowledge and attitudes towards childhood hearing loss among children aged 1–5 years with normal hearing sensitivity. While newborn hearing screening facilitates the early identification of congenital hearing loss, children may develop late-onset or progressive hearing loss that is not detected during the newborn period. Further, recurrent otitis media and other middle ear conditions are common during early childhood, which may adversely affect hearing and communication development. Developmental surveillance during these early years depends on timely recognition of communication milestones and early warning signs, maternal awareness of hearing loss and available intervention options is essential for timely identification and management. As mothers play a crucial role in the overall nurture and development of children, understanding their knowledge and attitudes towards childhood hearing loss is essential. Identifying existing gaps, barriers, and misconceptions will help develop and enhance awareness programs and campaigns, thereby improving overall outcomes for children. Therefore, the present study aimed to explore the knowledge and attitudes of mothers of children aged 1–5 years regarding childhood hearing loss, its risk factors, effects, and acceptance.

## Method

A cross-sectional survey was conducted after obtaining approval from the Institutional Ethics Committee of Kasturba Medical College, Mangalore. The study was conducted in two phases; phase one included the development and validation of the questionnaire, and phase two included data collection and analysis.

### Phase one – Questionnaire development, validation, and translation

Phase one involved the development and validation of the questionnaire, followed by translation into the local language, Kannada. The questionnaire was adapted from the mothers’ knowledge, attitudes, and practice towards Universal Newborn Hearing Screening measurement tool [21]. The original questionnaire [21] was designed to identify key concepts related to knowledge, attitude, and practices among mothers towards children with hearing loss and universal newborn hearing screening. The questionnaire was originally developed in the English language and later translated into Zulu by a professional translator. Permission was obtained from the primary authors for adaptation and use prior to the initiation of the study. The original questionnaire, designed for universal newborn hearing screening, was modified to accommodate the broader age range and objectives of the current study, which focuses on mothers of children aged 1–5 years. Questions pertinent to the newborn hearing screening were retained and additional items on late-onset hearing loss, middle-ear infections, amplification devices, rehabilitation services, and inclusion in school settings were added.

### Translation of the questionnaire

The adapted version was translated into the Kannada language, using the standard forward-back translation procedure [22,23]. The questionnaire was translated into Kannada by two native Kannada speakers who were fluent in English and could read and write Kannada. Among them, one was an audiologist and was aware of the constructs being measured by the questionnaire to ensure technical and clinical accuracy. The other was naïve about the construct and could provide an unbiased linguistic perspective. The Kannada versions were back-translated to English by two bilingual native Kannada speakers who were blinded to the original version. All forward and backward translations were carried out independently and without any assistance. The Kannada translations were compared and compiled to produce a final Kannada version. This final version was reviewed for readability, grammar, and language accuracy by a Kannada language expert with a PhD in Kannada literature and 10 years of experience.

### Content validity

The questionnaire was content-validated by three audiologists with more than five years of work experience in pediatric audiology, a master’s or PhD degree in Audiology, and proficiency in Kannada and English. The experts were asked to content-validate the questionnaire using a rating scale: 4 – relevant, 3 -quite relevant, 2 – somewhat relevant, 1 – not relevant. Items rated as relevant and quite relevant were retained. A Scale-Content Validity Index [24] was calculated to establish content validity. The author team reviewed and finalised the questionnaire for further use in the study.

### Face validity

The face validity of the questionnaire in Kannada was assessed with five Kannada-speaking mothers from the study site via convenience sampling. They were explained about the purpose of the study and asked if they would be willing to review the questionnaire. Those who accepted were provided with a consent form and a questionnaire in the Kannada language for review. A four-point scale (1- strongly disagree; 2 – disagree; 3 – agree, and 4 – strongly agree) was provided [25], and the respondents were asked to rate readability, familiarity with the content, and language. The questionnaire thus made was used for data collection in phase two. The questionnaire will be available on request from the authors.

### Phase two – Data collection

Data collection was conducted using a convenience sampling approach at the Department of Audiology and Speech-Language Pathology, Kasturba Medical College Mangalore, under the Manipal Academy of Higher Education, a tertiary care hospital serving the Dakshina Kannada district of Karnataka, India. The collection was carried out from 5^th^ June 2024–15^th^ January 2025.

The inclusion criteria were mothers of children aged between 1 and 5 years with normal hearing sensitivity, who were willing to participate, provided informed consent, and were able to read and write in Kannada to ensure accurate comprehension and completion of the questionnaire. For children, normal hearing sensitivity was confirmed through existing clinical audiological records and parental reports. The exclusion criteria excluded mothers of children diagnosed with hearing loss, any developmental disorders, or neurological conditions. Furthermore, mothers with an educational background in any healthcare profession were excluded as their knowledge could bias their responses. Mothers who could not read and write Kannada and who did not consent to participate were excluded.

The mothers were explained about the study, voluntary participation, confidentiality, and provided with a subject information sheet. Only those mothers who consented and signed the informed consent sheet were recruited and provided the questionnaire. The participants took approximately 12–15 minutes to complete the questionnaire.

### Statistical analysis

The Scale-Content Validity Index [24] was calculated to establish the content validity of the questionnaire. Descriptive statistics, including mean, standard deviation, and range, were used to summarise continuous variables, while discrete variables were summarised using frequency and percentages. The association between maternal educational levels and responses to items related to knowledge and attitudes was analyzed using Pearson’s chi-square test. Since the assumptions of the Pearson’s Chi-Square test were violated due to small, expected cell counts, Fisher’s Exact Test was used. A p-value of less than 0.05 was considered statistically significant. All statistical analyses were carried out using Jamovi software (version 2.6) [26].

## Results

### Content validity

A score of 0.85 was obtained for the Scale-Content Validity Index (Sc-CVI), indicating excellent content validity for the questionnaire.

### Face validity

The face validity assessment used a four-point scale, and all participants rated the questionnaire items as ‘agree’ or ‘strongly agree’. All of them indicated that the questionnaire instructions were clear. The questions and their response options were easy to understand.

A total of 215 mothers participated in the study, with a mean age of 29.35 ± 4.85 years, range 19–41 years. The mean age of the youngest child was 3.2 ± 1.2 years. Most mothers had 2 children (64.2%), while 14.4% had 1 child, 15.8% had 3 children, and 5.6% had more than 3 children. Regarding their educational status, 50.7% had completed high school, 33.1% had completed secondary schooling, 13.9% had completed primary education, and 2.3% had no formal schooling. The majority were homemakers (85.6%), while only 14.4% were employed. Most mothers (82.3%) indicated that their child had undergone hearing screening at birth, while 15.3% reported no screening was conducted, and 2.23% were unsure. Further, 79.1% mothers expressed concern about their child’s hearing and wanted more information.

### Knowledge regarding childhood hearing loss among the mothers

More than 60% of mothers demonstrated awareness of basic concepts related to childhood hearing loss. A vast majority (67%) indicated that a child can be born with hearing loss, while 23.3% responded no, while 9.8% were unsure. Similarly, 67.4% understood that a child can pass the hearing test at birth and later experience hearing loss, while 18.1% believed it was not possible and 14.4% were unsure. Similarly, 68.8% correctly responded that hearing loss can be detected in children at any age, while 18.6% and 12.6% responded no and unsure, respectively.

The mothers were asked to indicate which factors they believed could lead to hearing loss in young children. The two most identified factors were infection after birth (40.5%) and premature births (36.3%). Notably, 56 mothers (26%) reported being unsure about what could lead to hearing loss in children., Very few mothers (2.3%) attributed hearing loss to nonadherence to ancestral rituals, and none attributed it to nonadherence to birth rituals. Table 1 depicts the distribution of responses from mothers regarding potential causes of hearing loss in young children.

**Table 1:** Potential causes of hearing loss.

Potential causes of hearing loss	Percentage of responses n (%)
Infection after birth	87 (40.5%)
Premature babies	78 (36.3%)
Disease of the ear	73 (34%)
Infection during pregnancy	73 (34%)
Smoking, alcohol and drug abuse	67 (31.2%)
Chemical exposures	65 (30.2%)
Injury during delivery	49 (22.8%)
Use of traditional medicine	40 (18.6%)
Hereditary	25 (11.6%)
Emotional abuse	22 (10.2%)
Noise exposure	19 (8.8%)
Non-nutritional diet	11 (5.1%)
Bewitched/witchcraft	9 (4.2%)
Physical abuse	9 (4.2%)
Medications	9 (4.2%)
HIV positive pregnant mothers	8 (3.7%)
Nonadherence to ancestral rituals	5 (2.3%)
Nonadherence to birth rituals	0 (0%)

Note: More than one option could be selected.

The mothers were asked to indicate the treatment options for children with hearing loss. The top 2 notable options selected were specific procedures by doctors (96.7%) and medicines (74.9%). Table 2 presents the mothers’ responses to the treatment options for children with hearing loss.

**Table 2:** Treatment options for children with hearing loss.

Treatment options	Percentage of responses n (%)
Specific procedures by doctors	207 (96.7%)
Medicine	161 (74.9%)
Hearing aids/cochlear implants	91 (42.3%)
Prayers	14 (6.5%)
Cultural rituals	9 (4.2%)
Local herbs/treatments	8 (3.7%)
Traditional healers	7 (3.3%)

Note: More than one option could be selected.

More than 80% mothers recognized the observable signs, symptoms, and clinical confirmation as means of determining whether a child has hearing loss. Mothers correctly identified that a child with hearing loss will show no response to sound (92.6%) and delay in development (communication, speech, talking) (83.3%). Furthermore, 81.9% believed that doctors would inform parents if their child had hearing loss. Only 3.3% felt that it is not easy to know if a child has hearing loss.

**Fig 1:**
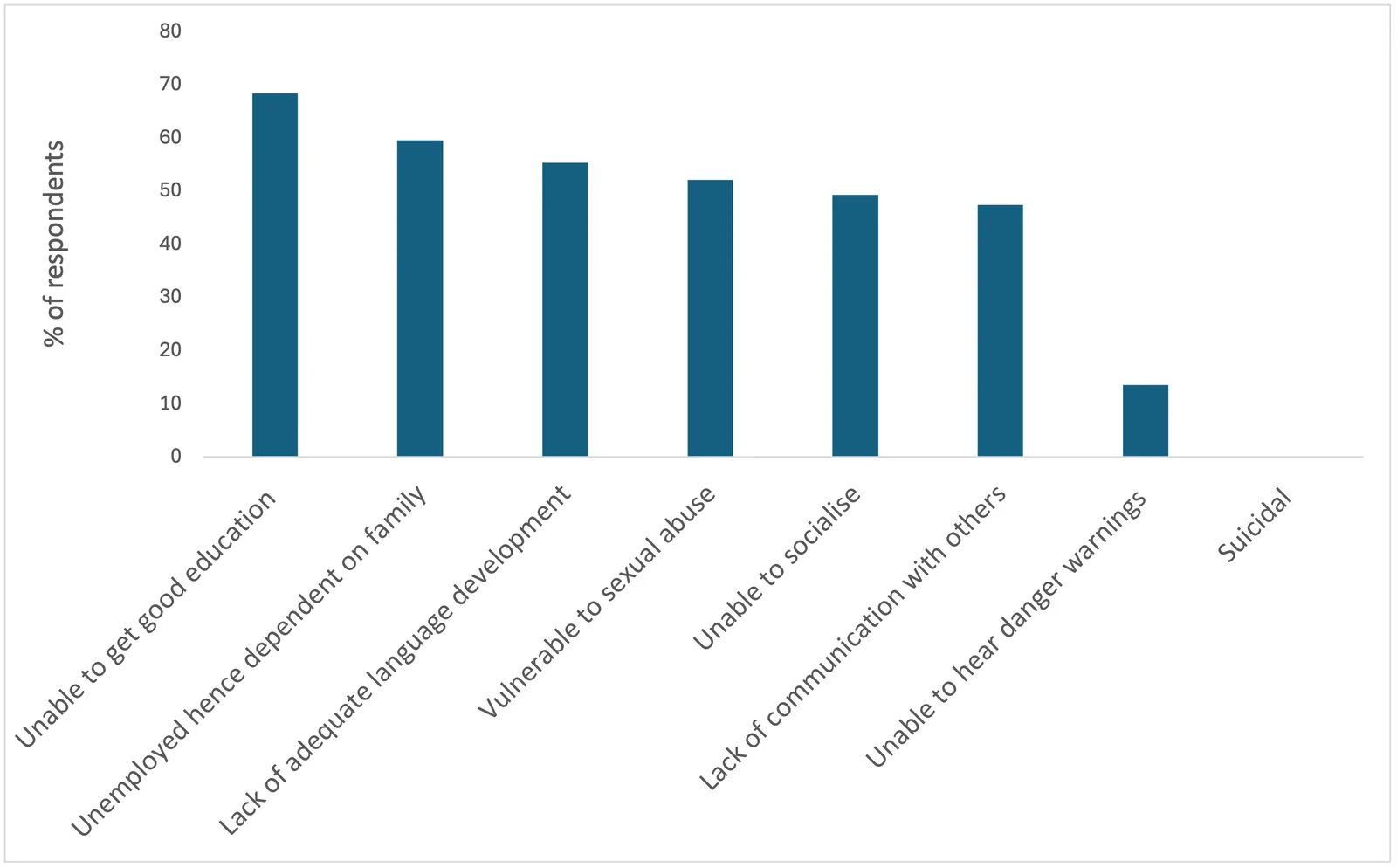
Respondents’ responses to effects of hearing loss.

The respondents were asked to identify, according to them, the effects of hearing loss. Only 2 mothers (0.9%) did not know any of the effects of hearing loss, while none of the mothers attributed it to having suicidal tendencies. Fig 1 depicts the distribution of responses.

Regarding awareness of middle ear infections in children, 60.5% reported being aware of these infections, while 71.6% believed they can be prevented. More than half of the respondents (58.6%) believed that the wrong positioning of a child while breastfeeding can lead to ear infection, while 20% disagreed, and 21.4% were unsure. The mothers were asked to identify the symptoms indicative of middle ear infections in children, 33% were unsure of any symptoms. The most recognized symptom was ear discharge (63.3%), followed by hearing loss and ear pain (58.6%) and fever (31.2%).

The association between maternal education levels and awareness of late-onset hearing loss, the possibility of a child being born with hearing loss, the ability to detect hearing loss at any age, middle ear infection in children and the prevention of ear infection was examined using Fisher’s Exact test. No statistically significant association was observed between educational level and responses to any of these questions (p = 0.69, p = 0.26, p = 0.19, p = 0.23, p = 0.06 respectively).

### Attitudes towards childhood hearing loss

**Fig 2:**
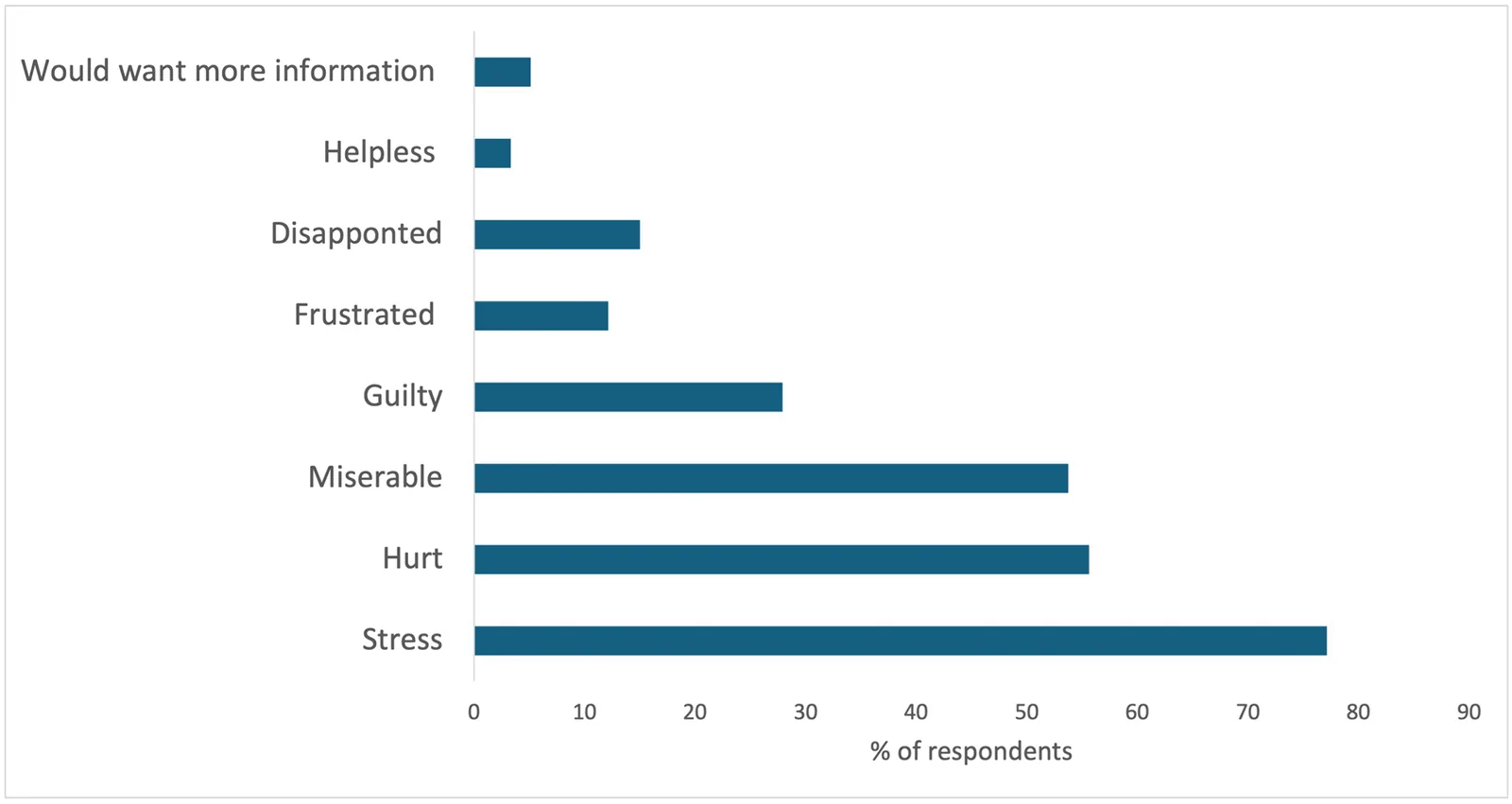
Respondents’ anticipated reactions to a diagnosis of hearing loss/deafness in their baby.

This section presents the mothers’ attitudes towards childhood hearing loss, focusing on their perceptions of hearing loss, identification and management, and reactions to the diagnosis. A vast majority (86.5%) would accept hearing screening for their children at different ages and at regular intervals, while 13.5% would not. Among those who did not accept, the reasons included ‘belief that hearing loss cannot happen to their child (2 mothers), while 1 mother selected ‘do not have enough information about hearing screening to make a decision’, and 26 respondents ‘did not have a reason’. Most mothers reported that they would have strong emotional reactions if their child were diagnosed with hearing loss, with the most being stressed (77.1%). Their responses have been depicted in Fig 2.

Regarding acceptance of amplification devices such as hearing aids or cochlear implants, only 27.4% (n = 59) showed acceptance of their child using these devices, while a majority (61.4%, n = 132) stated they would not, and 11.2% (n = 24) were unsure. A significant association was observed between maternal educational level and willingness to accept hearing aids/cochlear implants for their child (Fisher's Exact Test, p = 0.008). Acceptance rates varied across educational groups, with 33% (n = 36) of mothers with college education, 12.7% (n = 9) with high school education, 43.3% (n = 13) with primary education, and 20% (n = 1) of those who had not attended school reporting that they would accept hearing aids/cochlear implants for their child.

Most mothers (65.6%) did not believe children with hearing loss could attend regular school, 26.5% felt they could, and 7.9% were unsure. Similarly, 62.8% did not think children with hearing loss could have similar educational opportunities as those with normal hearing, 28.8% believed they could, and 8.4% were unsure. Significant associations were observed between maternal educational level and perceptions regarding the educational inclusion of children with hearing loss. Educational level was significantly associated with beliefs about whether children with hearing loss can attend regular schools (Fisher's Exact Test, p = 0.015) and whether they can achieve educational opportunities similar to those of children with normal hearing (Fisher's Exact Test, p = 0.001).

Regarding the impact of hearing loss on family and community, 69.7% felt it has a serious impact, 17.2% felt it has a somewhat serious impact, 9.8% rated it as very serious, and only 3.3% considered it not serious. Regarding the attitude of the community towards someone with hearing loss, more than half of the mothers (53.3%) felt that people are friendly, but they generally try to avoid the person. Further, 27% stated that most people pay no attention to the person, while 19.5% stated that the community usually supports & assists the person. Maternal educational level was not significantly associated with perceptions of community attitudes toward individuals with hearing loss (Fisher's Exact Test, p = 0.164).

### Sources and types of information

When asked what additional details they would like to obtain if their child is at risk for hearing loss, more than 90% wanted information on causes (93.5%) and treatment (92.6%). A large number (84.7%) wanted details on where to go for help, while 34% were interested in learning more about the expenses involved. The mothers were asked where they first learned about hearing loss. Among the sources, hospitals/clinics were the most popular choices (81.9%). Few mothers had learned about hearing from other sources such as newspapers (3.7%), family/friends/neighbours (3.3%), brochures and posters (2.8%). 8.4% reported not having heard about it. The final question asked the mothers their preferred resources for guidance on ear and hearing care and hearing loss in children. Their preferences have been depicted in Table 3.

**Table 3:** Resources for guidance on ear and hearing care and hearing loss in children.

Resources preferred for guidance	n (%)
Clinic/health workers	193 (89.8%)
Newspaper	131 (60.9%)
Brochures and posters	89 (41.4%)
Family/friends/neighbours	83 (38.6%)
Radio	60 (27.9%)
Television	22 (10.2%)

Note: More than one option could be selected.

## Discussion

The present study explored the knowledge and attitudes of mothers of children aged 1–5 years regarding childhood hearing loss. The findings revealed a low-to-moderate level of knowledge among mothers regarding childhood hearing loss and its effects. Nevertheless, gaps can be noted in the potential causes, risk factors, and treatment options, as well as in attitudes towards childhood hearing loss. The high percentage of mothers reporting that their child underwent a hearing screening at birth suggests high coverage of the newborn hearing screening program. Interestingly despite the inclusion of children with normal hearing sensitivity, 79% mothers expressed concerns regarding their child’s hearing and wanted additional information. This finding highlights the importance of providing parents with appropriate education regarding normal hearing and speech-language development. Further, as late-onset hearing loss, including hearing loss due to middle ear infections and other conductive causes, is common, parental counselling and follow-up education are important components of a successful early hearing detection and intervention program and should not be overlooked [10,27,28]. Addressing these through effective counselling and parental awareness programs will help in ensuring timely referrals and follow-up testing if warranted.

### Knowledge regarding childhood hearing loss among the mothers

Over 60% of mothers were aware of aspects such as the congenital nature of hearing loss, late-onset hearing loss, and the fact that it can be detected at any age. This level of awareness is comparable to findings from other similar settings and backgrounds [16,18,19] where parents often demonstrate basic awareness of childhood hearing loss but may lack detailed knowledge regarding its causes, risk factors, and management. Mothers in the present study commonly identified infections after birth, prematurity, ear diseases, and infections during pregnancy as potential causes of childhood hearing loss, which are consistent with established risk factors reported in the literature. These are consistent with the well-established medical causes of childhood hearing loss [6,29]. Although genetic causes account for almost 80% of prelingual hearing loss [30,31], only 11.6% identified hereditary factors. This finding highlights the need for greater public education regarding the genetic basis of hearing loss and the role of genetic counselling. This lack of awareness and under-recognition of genetic and hereditary causes could be due to limited literacy about genetics and limited access to genetic testing and counselling. Past studies have also highlighted a need to improve awareness among audiologists and speech therapists [32,33], as well as of the genetic basis of hearing loss, which in turn would improve public awareness.

Interestingly and encouragingly, just a handful of mothers attributed childhood hearing loss to beliefs such as witchcraft or nonadherence to ancestral rituals, and none of them attributed it to nonadherence to birth rituals. This contrasts with some of the earlier studies, where superstitious cultural beliefs related to hearing loss were commonly held [34–36]. 26% mothers reported not knowing any causes of hearing loss in children, which is alarming and highlights the need for having more awareness programs to focus specifically on this population.

Nearly all the mothers believed that hearing loss can be treated by following specific procedures recommended by doctors, while three-fourths believed medicines would help. Although this is suggestive of trust in the medical line of treatment, it could also be indicative of a misconception or lack of awareness, as fewer than half selected hearing aids or cochlear implants. Several forms of childhood hearing loss are permanent and cannot be reversed by medication alone [6,30]. Timely access to amplification devices and rehabilitative interventions enables optimal outcomes. The low proportion of mothers selecting hearing aids or cochlear implants may indicate gaps in awareness and understanding of amplification devices as reflected in their responses to attitude-related items. On the positive side, most correctly identified the observable signs and symptoms of hearing loss, such as lack of response and delay in speech development. Over 50% of respondents identified most of the effects of hearing loss. However, the more unobvious effects, such as being unable to hear danger signs and having suicidal tendencies, were missed by most.

Otitis media is the second most common diagnosis among children in the emergency department, and about 80% children experience it at least once in their lifetime [37,38]. Awareness of middle ear infections among mothers was moderate, with more than 60% aware of these infections and believing they can be prevented. While the majority of respondents correctly identified symptoms such as ear discharge, hearing loss and ear pain, one-third were unsure about these symptoms. Such gaps are concerning, as the timely recognition of early signs of otitis media plays an important role in facilitating timely consultation and treatment. Delays could lead to recurrent or chronic otitis media, which can cause conductive hearing loss and may adversely affect speech and language development in children. These findings indicate the need to target awareness initiatives to overcome these important knowledge gaps.

Interestingly, no significant association was found between maternal education level and awareness of congenital hearing loss, late-onset hearing loss, hearing loss detection, middle ear infection, or prevention of ear infections. These findings suggest that basic awareness of childhood hearing loss may be relatively consistent across educational groups and not solely dependent on formal education. As hearing loss is a hidden disability, a widespread sensitization program through healthcare professionals, digital media, and community health programmes may help to bring about a more uniform awareness among the population irrespective of educational background.

### Attitudes towards childhood hearing loss

Attitudes towards childhood hearing loss were largely positive, with over 85% willing to accept repeated screening at different ages. These findings are consistent with previous studies, which have shown a high acceptance among parents towards screening and diagnostic procedures [12,16,18]. Unfortunately, the attitudes towards rehabilitation procedures and inclusivity were negative. Only a quarter of respondents were willing to consider hearing aids or cochlear implants for their child, and most believed that children with hearing loss can attend regular school. Despite good awareness, such negative attitudes can hinder timely intervention and inclusivity, thereby impeding the long-term outcomes [39,40]. These discrepancies between higher acceptance of screening and lower acceptance of amplification devices may reflect misconceptions or concerns regarding amplification devices, visible assistive devices, financial constraints, cosmetic concerns, and cultural perceptions [15,21]. Parental stress and anxiety associated with hearing screening and diagnosis of hearing loss are commonly reported [41,42], highlighting the importance of family support.

An important finding of the present study was that maternal educational level was significantly associated with attitudes toward implication and management options for childhood hearing loss, despite showing no significant association with most knowledge-related items. Mothers with different educational backgrounds varied significantly in their willingness to accept amplification options such as hearing aids or cochlear implants for their child, and in their perceptions of the ability of children with hearing loss to attend regular schools and achieve educational opportunities comparable to those of their hearing peers. These findings suggest that education level may influence perceptions and possibly decision-making more strongly than basic awareness about hearing loss and its implications.

The low level of acceptance of amplification options such as hearing aids and cochlear implants, observed in the present study, is concerning. Only 27.4% of mothers indicated that they would accept the use of hearing aids or cochlear implants for their child if required. The finding suggest that awareness of hearing screening does not necessarily translate into acceptance of intervention. However, the present study did not specifically assess the reasons underlying mothers’ reluctance or refusal to accept these devices. Previous research indicates parental acceptance of amplification devices can be affected by perceived stigma, concerns about appearance, social acceptance, misconceptions, financial burden, emotional stress, and adjustment issues [43–45]. Other factors include service delivery challenges, social issues, time constraints, surgical considerations, and child-related factors [46]. Addressing these multifaceted barriers is crucial to improving parental acceptance and ensuring timely intervention for children with hearing loss.

In the present study, the majority of mothers did not believe that children with hearing loss could attend regular schools or access educational opportunities comparable to those of children with normal hearing. These findings are also concerning as substantial evidence indicates that children with hearing loss who receive timely identification and intervention can successfully participate in mainstream education and have favourable outcomes. Yoshinaga-Itano et al. [47] reported that early detection and intervention are associated with improved language and educational outcomes. In contrast, Moeller [48] reported significantly better language development among children enrolled in early-intervention programs. Addressing these misconceptions through parent education and counselling is essential to promote positive attitudes and facilitate better decision-making by mothers regarding academic education options.

### Sources and types of information

More than 80% mothers expressed an interest in learning about the causes of hearing loss, treatment options, and where to seek help. These are similar to the areas needing more awareness programs identified by the present study among mothers of children aged 1–5 years, as well as by previous studies among mothers of infants [9,16,28,34,35,49].

Clinics and health workers were the most preferred sources of information, highlighting the crucial role of healthcare services in providing education on ear and hearing care.. These are consistent with the guidelines and recommendations of integrating ear and hearing care education with primary health services to improve awareness levels [50].

Collectively, the findings indicate that although awareness of childhood hearing loss was generally comparable, notable differences existed in attitudes. These findings highlight the need for public health initiatives that go beyond knowledge dissemination and specifically address misconceptions about amplification devices, academic opportunities, and the attitude towards children with hearing loss. Parent-focused counselling and educational programs may be instrumental in improving acceptance of rehabilitation options, fostering positive attitudes toward inclusive education, and enhancing expectations regarding the long-term developmental, educational, and social outcomes of childhood hearing loss and its implications.

### Limitations and future recommendations

Some limitations of the present study should be acknowledged. As the questionnaire was designed to assess multiple domains of knowledge and attitudes rather than a single underlying construct, internal consistency measures such as Cronbach's alpha could not be estimated. The test-retest reliability could not be tested as the participants were not available for a follow-up assessment after completion of the initial survey. The use of a self-reported questionnaire may have introduced recall bias for knowledge items and social desirability bias for attitude items. Participants were recruited via convenience sampling from a single tertiary care hospital, introducing some selection bias. Their knowledge and attitude levels might be differ from those of the general population, limiting generalizability to other settings. Next, the cross-sectional study design limited the ability to establish causal relationships between the variables. Recruitment from a tertiary care centre may have resulted in a sample with greater exposure to hearing health information than the general population, potentially limiting the generalizability of the results. Further, the study population included only mothers and excluded fathers and other caregivers, who play a crucial role in decision-making for young children. The questionnaire primarily assessed awareness levels and self-reported knowledge, which may not fully capture the depth or accuracy of participants’ understanding of childhood hearing loss and its management.

Future efforts should prioritize strengthening parental education regarding childhood hearing loss, focusing on its causes, risk factors, signs, and the importance of early identification and rehabilitation. Educational and awareness initiatives are needed for widespread outreach through clinics, hospital settings, schools, and community-based platforms. At the policy level, initiatives focusing on promoting inclusivity, improving access to hearing healthcare services, and developing and strengthening community-based programs are essential to reduce stigma and address prevailing misconceptions. Further, longitudinal studies involving families of children diagnosed with hearing loss and those of children with normal hearing, are recommended to assess changes in knowledge and attitude over time to understand long-term effectiveness and impact better. Future studies should use probability-based sampling methods and include participants from community and primary healthcare settings to enhance the generalizability of the findings.

## Supporting information

S1 Data Data sheet. (CSV)
